# 

**Published:** 1882-08

**Authors:** 


### INDEX.

### A.

PAGE.

Acne, Treatment of ... 23
Alumni Association of the Buffalo Medical College ... 380
American Medical Association ... 565
Antisuppurative, Sulphide of Calcium as an ... 32
Antiseptics in Typhoid Fever ... 507
Andrews, Judson B., M. D., Some Practical Remarks upon the Subject of Insanity ... 586
Aphasia ... 529
Arsenical Poisoning, Dialyzed Iron in the Treatment of ... 21
Arsenical Poisoning, Unusual Post Mortem Appearances in ... 117
Ashhurst, John Jr., M. D., on Gangrene of the Leg ... 433
Auscultation, an Interesting Question in, by P. W. Van Peyma, M.D. 270

### B.

Bartow, Bernard, M. D., on Spinal Extension by Suspension ... 1
Bartow, Bernard, M. D., on Erysipelas Occuring with Parturition ... 319
Baker, F. J., M. D, on the Present Status of Medicine and its Relation to the Commonwealth in the Future ... 7
Beane, Frank Dudley, A. M., M. D., on Histology of the Nerves ... 97

PAGE.

Beane, Frank Dudley, A. M., M.D., Histology of the Nerves ... 145
Beane, Frank Dudley, A. M., M. D., Urethral Stricture, a Clinical Contribution to Treatment of, by ... 207
Bladder, New Way to Detect Stone in the ... 88
Brandy, Adulteration of ... 554
Buffalo Medical and Surgical Association, Regular Meeting, March 6th, 1883 ... 418
Buffalo Medical and Surgical Association ... 323
Buffalo Medical and Surgical Association ... 276
Buffalo Medical and Surgical Association, Regular Meeting, February 6th, 1883 ... 366
Buffalo Medical and Surgical Association ... 130
Buffalo Medical and Surgical Association ... 555
Buffalo Medical College, Session of 1882–83 ... 141
Buffalo Medical and Surgical Association, Regular Meeting, October 2d. 1882 ... 178
Buffalo Medical and Surgical Association, Regular Meeting, November 6th, 1882 ... 231
Buffalo State Asylum for Insane, Annual Report ... 136

### C.

PAGE.
Cancer of the Pylorus, a Clinical Lecture on, by Thomas F. Rochester, M. D. .... 152
Cancer, Billroth on Removals of... 33
Charter of the College of Physicians and Surgeons.... 349
Changes of Uterine Muscular Tissue after Parturition.... 418
Chorea, Cardiac Symptoms of..... 87
Clark, Alonzo, M. D., on Bright's Disease.... 123
Convulsions in Children.... 546
Correspondence.... 499
Correspondence.... 453
Correspondence.... 413
Correspondence.... 322
Correspondence.... 270
Correspondence.... 224
Code, Discussion on the New..... 90
Consultation Question, the Prevailing Sentiment on the.... 186
Commencement, the Medical..... 382

### D.

Daggett, B. H., M. D., on a Case of Diffuse Traumatic Cellulitis...... 450
Davidson, A.R., M.D., on Arsenical Poisoning.... 117
Davidson, A.R., M.D., Letters from Philadelphia ....224, 270
Davidson, A.R., M.D., Letters from London ....414, 453, 499
Disinfectants, Koch on.... 175
Diabetes, Malaria and.... 19
Diabetes, Iodoform in.... 89
Doveri, Pulvis.... 223

### E.

Editorial Notes.... 142
Editorial Notes.... 187
Editorial Notes.... 285
Editorial Notes.... 333
PAGE.
Epileptic Attacks, Albuminuria as a Symptom of.... 89
Ergot in Skin Diseases.... 512
Erie County Medical Society ..... 327
Erie County Medical Society ...... 557
Erysipelas, White Lead Paint in... 519

### F.

Fistula Knife .... 177
Formad, Dr., on Examination of Urine.... 553
Frederick, C. C., M. D., Ergot in Obstetrics, the Abuses of, by.... 198

### G.

Gay, Charles C. F., M. D., an Inquiry into the Source and Cause of Secondary or Recurrent Hemorrhage after Ligation of Arteries for Aneurism.... .. 159
Gay, Charles C. F., M. D., Tonsillotomy.... 446
Goodell, William, M. D., Cancer of the Neck of the Womb.... 289
Graves' Disease.... 129
Granger, Wm. D., on Paresis ..... 537
Glycerine .... 464

### H.

Hamilton, Prof. F H., Letter from, on Sewer Gas and its Dangers.... 37
Hayd, Herman, M.R., C.S., England, Scirrhous Cancer of Rectum..... 497
Hemorrhoids, a New Treatment for. Reported by Thomas Lothrop, M. D.... 219
Hopkins, H. R., M. D., Case Illustrating the Importance of Diagnosis .... 14
Hopkins, H. R., M. D., on Aphasia, 529
Hot Water in Therapeutics........ 513

PAGE.

Howe, Lucien, M. D., on the Electro-Magnet in Removing Particles of Iron from the Eye............ 345

I.

Inebriety, Bromides in............ 18
Infancy, Narcotism in............ 167
Institutions, Our Public............ 232
Insanity, Some Practical Remarks upon the Subject of, by Judson B. Andrews, M. D................ 386
Iodoform, to Cover the Odor of... 90
Iodoform, the Odor of ........... 176

K.

Kidneys, Floating................ 174

L.

Laryngeal Phthisis, Hydrastin in.. 322
Law and Malpractice ............. 282
Leucorrhœa in Children........... 550
Legislation, Erie County Society Committee on.................. 566
Library Association, the Buffalo Medical...................... 281
Library Association, the Buffalo Medical...................... 368
Library Association, the Buffalo Medical..................... 284
Ligation, Hemorrhoids Treated by. 440
Lothrop, Thomas, M. D., on a New Treatment for Hemorrhoids..... 219
Lynde, U. C., M. D., Hemorrhage from the Navel after Detachment of the Funis................... 411

M.

Malignant Tumors, Some Points on the Histology of, by M. D. Mann, M. D.................. 300
Malaria in Skin Diseases.......... 229
Male Fern, Oleoresin of.......... 511

PAGE.

Mann, M. D., M. D., on Some Points on the Histology of Malignant Tumors.................. 300
Mann, Mathew D., A. M., M. D., Abortion, the Management of... 49
Medical Examiners, State Board of, 373
Medical Club, Meeting of the Buffalo.......................... 66
Medical Corps, U. S. Army....... 463
Moody, Mary B., M. D., Bacillus Tuberculosis.................... 352

N.

New Code, the Erie County Medical Society and the............. 332
New Medical Code...... ....... 517
Neuralgia and its Treatment, Practical Notes on.................. 29
Nitrous Oxide and Chloroform.... 464
Nurses, Directory for............. 331

O.

Opium, Inebriety and the Use of.. 173

P.

Paralysis, Facial.................. 126
Paresis, or General Paralysis of the Insane......................... 537
Pepper, William, M. D., LL. D., Intra-Cranial Tumor............ 481
Pepper, William, M. D., LL. D., on Calculous Pyelitis........... 241
Peterson, Frederick, M. D., on Contagium Animatum.............. 489
Peterson Frederick, M.D., Letter from Strasburg............. ... 34
Phthisis Pulmonalis, Hypophosphites in....................... 176
Pilocarpin, An Experimental Study on the Action of, by Dr. Julius Pohlman....................... 441

PAGE.
Pohlman, Dr. Julius, on an Experimental Study of the Action of Pilocarpin........................ 441
Provident Dispensary and Hospital Association.................... 383
Pryor, J. H., M. D., on Excessive Masturbation in the Female, with an Unusual Method of Performance............................ 62
Public, the Profession or the...... 475
Pyrogallic Acid, Treatment of Phagedæna.................... 321
Pryor, J. H., M. D., on Two Cases of Simple Fracture of the Skull Unrecognized for Days ......... 119

### R.

Radical Cure of Hernia........... 462
Reduction of Dislocated Femur... 518
Rochester, Thomas F., M. D., a Clinical Lecture on Cancer...... 152

### S.

Seguin, E. C., on Fowler's Solution, 169
Sick Headache.................. 222
Specialist, Titles of............... 177
Spinal Sclerosis.................. 127
State Board of Medical Examiners, 563
Stockton, Charles G., M. D., Oxaluria, Something About.......... 57
Stockton, Chas. G., M. D., Ergot in Obstetrics, the Uses of........... 193
Strabismus Convergens, the Management of..................... 516
Syphilitic Re-infection of Husband and Wife......... ............. 510
Syrup, Amussat's Laxative......... 177

### T.

PAGE.
Thomas, T. G., M. D., on Infantile Leucorrhœa................. ... 550
Tremaine, W. S., M. D., Antiseptic Surgery........................ 337
Tremaine, W. S., M. D., U. S. A., on Physiological Action of Spinal Accessory Demonstrated by Injury of its Filaments............ 218
Tremaine, W. S., M. D., U. S. A., Clinical Lecture Delivered at the Hospital of the Sisters of Charity, Jan. 13, 1883............. ... ..307

### U.

University of Buffalo.............. 377
Urine, Rules for Examination of... 553

### V.

Van Peyma, P. W., M. D., on Complete Placenta Praevia.......... 16
Van Peyma, P. W., M. D., Conservative Surgery............... 121
Van Peyma, P. W., M. D., on an Interesting Question in Auscultation .......................... 270
Vaccination, Traumatic Tetanus, and Death from................. 174
Veins, Varicose.................... 175
Venereal and Common Warts...... 512

### W.

Wall, Chas. A., M. D., Burns and their Treatment................ 157
White Lead Paint in Erysipelas .... 519
Wise, P. M., M. D., Responsibility of the Examining Physician of the Insane..................... 403

